# Commentary: Acute Hemorrhagic Leukoencephalitis (Weston–Hurst Syndrome) in a Patient with Relapse-Remitting Multiple Sclerosis

**DOI:** 10.3389/fimmu.2016.00207

**Published:** 2016-05-25

**Authors:** Tatiana Koudriavtseva

**Affiliations:** ^1^Multiple Sclerosis Clinical and Research Unit, Department of Systems Medicine, Tor Vergata University, Rome, Italy

**Keywords:** interferon-β, multiple sclerosis, acute hemorrhagic leukoencephalitis, tumefactive demyelination, hemorrhage

Recently, Yildiz et al. reported the occurrence of acute hemorrhagic leukoencephalitis (AHLE) in a patient with pluriannual relapse-remitting multiple sclerosis (MS) after 1 month of high-dose interferon-β-1a treatment. The authors raised the question whether this case may be a hyperacute form of acute disseminated encephalomyelitis (ADEM) or a rare hemorrhagic evolution of tumefactive demyelination (TD), both unusual in the course of MS (1). They concluded that AHLE occurrence was unrelated to the underlying MS since hemorrhage has not been found in neuropathological patterns of both MS and TD. Although two published case reports of TD with hemorrhage in the course of fingolimod and natalizumab therapies (brain biopsy was not performed) have been mentioned in the paper, the potential predisposing effect of interferon-β therapy on the AHLE development has not been hypothesized.

However, since the symmetrical basal ganglia hemorrhagic lesions in this case were developed after the first month of high-dose interferon-β-1a treatment (1), the therapy influence should be taken into account. There were previously reported brain hemorrhages during treatment with both interferon-β in MS and interferon-α in HCV hepatitis (2), as well as a case of TD in pediatric MS patient under interferon-β therapy (3). Moreover, a thrombotic thrombocytopenic purpura with thrombotic microangiopathy and hemolytic–uremic syndrome has been recently included as a rare interferon-β adverse event in its package leaflet by the EMA. We also found in a cohort of 187 MS patients that a mild thrombocytopenia was more frequent (*p* = 0.02) in patients on high-dose interferon-β therapy (16%) compared to those on treatment with low-dose interferon-β (3.1%), other drugs (5%), including glatiramer acetate (4%), natalizumab (9.1%), and fingolimod (0%), or untreated patients (2.2%) (4). High-dose interferon-β therapy was associated with more than eightfold increase in the risk for thrombocytopenia (odds ratio 8.60, 95% confidence interval: 1.01–74.48).

The type I interferon-α/β rapidly produced during infection has antiviral activity and plays a key role in innate immunity, which represents the non-specific defense against infections and tissue damage acting through its essential processes including inflammation and blood coagulation (5). Thrombocytopenia is a frequent complication of viral infections, thus, we supposed that the interferon-activated innate inflammatory–thrombotic processes could lead to the increased platelets consumption. Moreover, it was recently demonstrated that mice treated with polyinosinic:polycytidylic acid, strong interferon-β inducer, showed thrombocytopenia and platelet hemostatic and inflammatory dysfunction, associated with abnormal megakaryocytes distribution in the bone marrow niches and directly correlated with the plasmatic and bone marrow interferon-β levels (6).

Indeed, platelets cooperate not only in hemostasis and thrombosis but also in inflammation, immunity, and tissue repair creating a link between innate and adaptive immunity (7). Platelet-activating factor (PAF), secreted by the cooperation of platelets and leukocytes, is a potent platelet activator and chemotactic stimulant for inflammatory cells and contributes to the early breakdown of the blood–brain barrier (BBB) through endothelial junction disruption (7). The chronic platelet activation is well established in MS, and PAF receptors are upregulated in MS lesions (7). Moreover, the release of adhesion molecule PECAM-1 to the circulation and associated leukocyte infiltration are likely consequent to platelet interaction with leukocytes at BBB endothelium (7). Furthermore, activated platelets amplify coagulation, whereas activated coagulation stimulates platelets (7). Recent experimental evidences of the coagulation cascade contribution in inflammatory, degenerative, and repair processes in the central nervous system (CNS) further support the previous data on the presence of several coagulation factors in MS lesions (8).

In the AHLE case described, a MRI-guided biopsy showed some partially thrombosed vessels with perivascular hemorrhagic necrosis, severe edema, and prevalently neutrophil inflammatory infiltrate, although a thrombocytopenia was not reported (1).

Since ADEM and AHLE are frequently preceded by infections, Yildiz et al. commented that the cross-reactivity between human myelin antigens and viral or bacterial antigens likely induce an excessive immunological response and mentioned an AHLE murine model determined by a strong activation of CD8+ T cells in C57BL/6 mice leading to hemorrhagic demyelination within 24 h following injecting of the VP2121–130 viral capsid peptide of the Theiler’s murine encephalomyelitis virus (1). In this murine model, a determinant role of hematopoietic factors in the onset of brain microhemorrhage and vascular permeability was subsequently showed because hemorrhage susceptibility of C57BL/6 mice was transferable with their bone marrow to hemorrhage-resistant 129 SvIm mice having indistinguishable CD8+ T-cell responses (9). Megakaryocytes producing platelets could be one among the others of these hematopoietic factors. Furthermore, injection of interferon-α/β was found to prolong the proliferation and expansion of antigen-specific CD8+ T cells during cross-priming, representing an additional mechanism by which the innate response to infection promotes adaptive immunity (10).

Undoubtedly, cerebral hemorrhagic lesions under or without interferon therapy are a very uncommon event in MS, likely determined by an exceptional coincidence of local and/or systemic predisposing conditions (probable concomitant infections, etc.). However, using T2*-weighted 3-T MRI, Zivadinov et al. have found a significant higher frequency of cerebral microbleeds in 445 MS patients compared to 177 healthy controls, particularly in subjects ≥50 years of age (*p* = 0.01, 19.8% vs. 7.4%, respectively) but similar to that of 51 patients with other neurological diseases (11). This finding further supports the essential role of inflammation and coagulation, both arms of innate immunity, in many if not all CNS inflammatory degenerative diseases approaching them in common baseline pathogenetic mechanism. The interferon-β activating the different patterns of innate immunity could lead in particular conditions to increased inflammatory–thrombotic processes up to hemorrhagic manifestations in the CNS.

## Author Contributions

The author confirms being the sole contributor of this work and approved it for publication.

## Conflict of Interest Statement

TK reports consulting fees from Bayer Schering, and Institutional grant from Merck Serono, Biogen Idec, Novartis, and Bayer Schering outside the submitted work.
